# Comparison of Demand for Drugs Used for COVID-19 Treatment and Other Drugs During the Early Phase of the COVID-19 Pandemic in Italy

**DOI:** 10.1001/jamanetworkopen.2020.37060

**Published:** 2021-02-08

**Authors:** Adriana Ammassari, Aurora Di Filippo, Maria Paola Trotta, Giuseppe Traversa, Andrea Pierantozzi, Francesco Trotta, Nicola Magrini

**Affiliations:** 1 Italian Medicines Agency, Rome, Italy

## Abstract

This cross-sectional study compares demand for drugs for treatment of coronavirus disease 2019 (COVID-19) between the period directly before the COVID-19 outbreak in Italy and the early period of the outbreak.

## Introduction

In February 2020, Italy was the first European country to detect coronavirus disease 2019 (COVID-19) in individuals and rapidly turned into one of the most-affected regions of the world. The National Health Service (NHS), which provides universal coverage to citizens, was challenged as never before in the history of the institution. Because no approved drug was available, patients received potentially effective drugs, participated in clinical trials, accessed compassionate drug use programs, or self-medicated.^1^ The aim of this study was to evaluate changes in drug demand during the early phase of the COVID-19 outbreak in Italy compared with the period before the outbreak.

## Methods

In this cross-sectional study, demand for medication was compared between the COVID-19 period (March to May 2020) and the pre–COVID-19 period (December 2019 to February 2020). These drugs included those used for COVID-19 that were allowed by the Italian Medicines Agency (hydroxychloroquine, lopinavir-ritonavir or darunavir-cobicistat, and low–molecular weight heparin), not allowed by the Italian Medicines Agency (azithromycin, immunomodulatory agents), and evaluated in clinical trials (eg, anakinra, colchicine) (eTable in the Supplement). Hospital-used injectables and the most frequently purchased over-the-counter drugs were also included in the comparison. Drugs used in a pharmaceutical-sponsored compassionate program were not considered (ie, remdesivir). Ethics committee approval was not required according to Italian legislation because the study was a descriptive analysis of aggregated data. This study followed the Strengthening the Reporting of Observational Studies in Epidemiology (STROBE) reporting guideline.

Nationwide non–diagnosis-linked administrative databases provided routinely collected data on drug demand for in-hospital use of drugs covered by the NHS, drugs dispensed by community pharmacies that were covered by the NHS, and drugs dispensed by community pharmacies through out-of-pocket purchase. The absolute difference in mean monthly demand per 100 000 population per day between the COVID-19 and the pre–COVID-19 periods (ADPP) was compared using a 2-sided *t* test. Analyses were performed using SAS, version 9.4 (SAS Institute). *P* = .05 was considered statistically significant. The relative difference in demand for drugs between the COVID-19 and pre–COVID-19 periods (RDPP) was used to describe between-period percentage change.

## Results

Compared with the pre–COVID-19 period, during the outbreak, public hospitals had significantly increased demand for the following drugs: azithromycin, hydroxychloroquine, tocilizumab, darunavir-cobicistat, anakinra, lopinavir-ritonavir, baricitinib, and sarilumab (Table). Mean (SE) RDPPs were highest for hydroxychloroquine (4661.67% [8361.01%]) and azithromycin (195.40% [229.45%]). Although back-shifting of hydroxychloroquine from hospitals to central distributors in June 2020 was recorded, sensitivity analysis confirmed these study results.

**Table.** Differences in Drug Demand During the Pre–COVID-19 and COVID-19 Periods

Drugs	Monthly packs per 100 000 population per day, mean (SD)		Absolute difference^a^		Relative change, % (SE)
	Pre–COVID-19 period	COVID-19 period	Monthly packs per 100 000 population per day, mean (95% CI)	*P* value	
Public hospitals					
Azithromycin	3.88 (1.04)	11.45 (7.44)	7.57 (4.45 to 10.69)	<.001	195.40 (229.45)
Hydroxychloroquine	0.13 (0.04)	6.14 (2.65)	6.01 (3.78 to 8.241)	<.001	4661.67 (8361.01)
Tocilizumab^b^	0.38 (0.11)	0.59 (0.36)	0.21 (0.12 to 0.29)	<.001	54.80 (18.06)
Darunavir-cobicistat	0.40 (0.07)	0.52 (0.32)	0.12 (0.07 to 0.17)	<.001	29.42 (18.11)
Anakinra	0.13 (0.04)	0.23 (0.11)	0.10 (0.03 to 0.17)	.01	73.80 (17.74)
Lopinavir-ritonavir	0.05 (0.04)	0.09 (0.11)	0.04 (0.02 to 0.07)	.003	97.64 (209.87)
Baricitinib	0.19 (0.07)	0.23 (0.07)	0.03 (0.01 to 0.06)	.003	17.46 (27.03)
Sarilumab	0.05 (0.01)	0.08 (0.03)	0.03 (0.01 to 0.04)	.001	60.10 (17.11)
Colchicine	0.02 (0.02)	0.04 (0.30)	0.02 (−0.001 to 0.04)	.057	70.36 (34.69)
Ruxolitinib	0.21 (0.05)	0.22 (0.05)	0.01 (−0.01 to 0.04)	.20	6.72 (5.14)
Canakinumab	0.03 (0.01)	0.04 (0.01)	0.01 (−0.01 to 0.03)	.16	45.03 (19.44)
Tofacitinib	0.08 (0.02)	0.08 (0.02)	0.01 (−0.23 to 0.24)	.95	8.44 (6.83)
Corticosteriods	24.68 (6.69)	24.37 (3.53)	−0.31 (−0.97 to 0.35)	.17	1.25 (22.14)
Heparin	49.05 (10.86)	46.16 (13.38)	−2.89 (−8.70 to 2.92)	.33	−5.90 (25.34)
Community pharmacies					
Drugs covered by the NHS					
Hydroxychloroquine	7.04 (0.11)	9.52 (0.21)	2.48 (2.20 to 2.75)	.001	35.15 (1.44)
Colchicine	0.90 (0.01)	0.88 (0.05)	−0.02 (−0.08 to 0.04)	.96	−1.99 (0.44)
Heparin	33.63 (0.93)	28.34 (1.15)	−5.29 (−7.01 to −3.57)	.02	−15.73 (3.28)
Azithromycin	35.36 (0.04)	26.74 (4.01)	−8.62 (−13.28 to 3.97)	.06	−24.39 (5.96)
Out-of-pocket purchase					
Anxiolytics	232.16 (25.52)	241.05 (24.62)	8.89 (6.34 to 11.44)	<.001	3.83 (4.07)
Hydroxychloroquine	6.80 (0.61)	14.41 (2.71)	7.61 (1.50 to 13.72)	.02	111.84 (601.64)
Vitamin D and analogues	160.45 (21.29)	165.12 (12.78)	4.67 (1.56 to 7.78)	.003	2.91 (1.91)
Ascorbic acid	1.60 (0.49)	2.15 (2.63)	0.55 (0.22 to 0.88)	.001	34.12 (15.58)
Drugs used for erectile dysfunction	27.93 (2.14)	17.49 (2.71)	−10.44 (−11.14 to −9.75)	<.001	−37.38 (6.74)
NSAIDs and antipyretics	709.02 (130.57)	600.14 (248.84)	−108.88 (−110.93 to −96.30)	<.001	−15.36 (30.51)

Abbreviations: COVID-19, coronavirus disease 2019; NHS, National Health Service; NSAIDs, nonsteroidal anti-inflammatory drugs.

^a^ Shown in decreasing order.

^b^ Only the intravenous formulation.

Similarly, requests of hospital-used injectables increased for muscle relaxants (RDPP [SE], 264.1% [218.3%]; ADPP, 4.93 [95% CI, 3.86-5.60]; *P* < .001), general anesthetics (RDPP [SE], 116.0% [39.4%]; ADPP, 9.49 [95% CI, 6.10-12.89]; *P* < .001), adrenergic and dopaminergic agents (RDPP [SE], 37.8% [82.3%]; ADPP, 0.59 [95% CI, 3.29-8.55]; *P* < .001), ascorbic acid (RDPP [SE], 204.5% [1719.0%]; ADPP, 0.46 [95% CI, 2.74-6.45]; *P* < .001), hypnotics and sedatives (RDPP [SE], 145.8% [203.7%]; ADPP, 4.57 [95% CI, 2.84-6.30]; *P* < .001), antidotes (RDPP [SE], 69.7% [841.4%]; ADPP, 1.34 [95% CI, 0.82-1.87]; *P* < .001), and antithrombotic agents (RDPP [SE], 27.6% [10.1%]; ADPP, 0.30 [95% CI, 0.09-0.52]; *P* = .007). The Figure shows the time series of percentage variation in monthly purchases for each drug smoothed by a 3-month moving average filter and indexed against January 2019.

**Figure.**
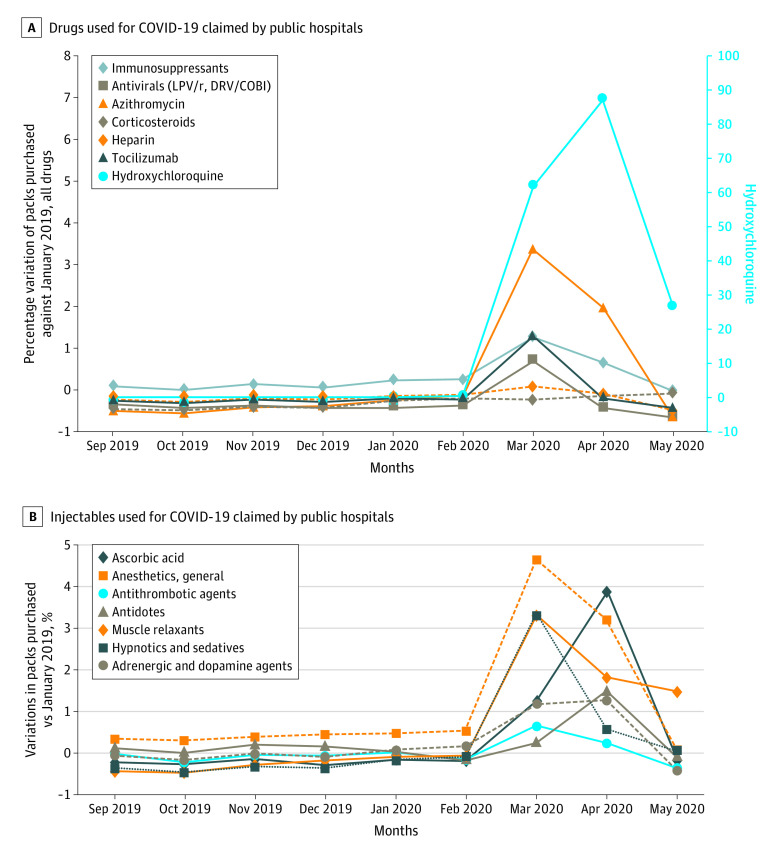
Drugs and Injectables Used for Coronavirus Disease 2019 (COVID-19) Claimed by Public Hospitals Drugs with the largest absolute change in demand are shown. B, Data shown are the 3-month moving average of the percentage difference compared with the fixed base of January 2019. Antivirals included darunavir-cobicistat and lopinavir-ritonavir.

Demand for COVID-19 drugs in community pharmacies paralleled in-hospital use trends. Although nonprescription requests for anxiolytics and vitamin supplements increased during the COVID-19 period, claims for erectile dysfunction drugs decreased (Table).

## Discussion

During the early phase of the COVID-19 outbreak in Italy, the Italian NHS experienced unexpected and exceptional changes in demand for drugs used for treatment of COVID-19 and injectables for supportive care. Increased claims for hydroxychloroquine is in agreement with US prescription refill patterns.^2,3^ Although the association of increased use with public expenditure was negligible, there may be ethical issues associated with off-label emergency use. More than granting drug prescriptions with an uncertain risk-benefit balance, participation of individuals in clinical trials should be promoted.^4^ In hindsight, 3 treatment scenarios can be identified. In phase 1 (March 2020), there were exponential increases in cases of COVID-19 and urgent requests for treatments with a small evidence base. In phase 2 (April 2020), the COVID-19 growth rate slowed and randomized clinical trials were implemented to provide results for better evidence-based practice. In phase 3 (May 2020), the epidemic curve was flattened and COVID-19 treatment approaches were reshaped on the basis of preliminary trial results.

Limitations of the study were that the analyzed pharmaceutical demand describes the NHS burden but does not comprehensively represent the Italian COVID-19 health care scenario (eg, compassionate use programs and clinical studies sponsored by pharmaceutical companies were excluded a priori) and tracking of medicines through the supply chain did not necessarily correspond to drug prescriptions. Out-of-pocket drug purchases revealed the association of the COVID-19 pandemic with the well-being of the general population. Risk of self-medication and panic buying is concerning.^5^ The results of this study underscore the importance of routine drug utilization monitoring as a useful tool to timely record quantitative and qualitative changes in demand for drug prescriptions.^6^

## Notes

Supplement. **eTable.** Drug Groups Included in the Study
