# Supplementary material for: Comparison of Demand for Drugs Used for COVID-19 Treatment and Other Drugs During the Early Phase of the COVID-19 Pandemic in Italy
Source: JAMA Netw Open. 2021 Feb 8;4(2):e2037060. doi: 10.1001/jamanetworkopen.2020.37060 (PMC7871193; doi:10.1001/jamanetworkopen.2020.37060)
Supplement: Supplement. — eTable. Drug Groups Included in the Study [file jamanetwopen-e2037060-s001.pdf]

# Supplemental Online Content

Ammassari A, Di Filippo A, Trotta MP, et al. Comparison of demand for drugs used for COVID-19 treatment and other drugs during the early phase of the COVID-19 pandemic in Italy. *JAMA Netw Open.* 2021;4(2):e2037060. doi:10.1001/jamanetworkopen.2020.37060

**eTable.** Drug Groups Included in the Study

This supplemental material has been provided by the authors to give readers additional information about their work.

© 2021 Ammassari A et al. *JAMA Network Open.*

eTable. Drug Groups Included in the Study

| Indication | Category | WHO's ATC | | | | | | | | |
|---|---|---|---|---|---|---|---|---|---|---|
| Drugs for COVID-19 treatment | Azithromycin | J01FA10 | | | | | | | | |
| Drugs for COVID-19 treatment | Hydroxychloroquine | P01BA02 | | | | | | | | |
| Drugs for COVID-19 treatment | Corticosterioids | H02AB02, H02AB04 | | | | | | | | |
| Drugs for COVID-19 treatment | Tocilizumab | L04AC07 | | | | | | | | |
| Drugs for COVID-19 treatment | Darunavir/ cobicistat | J05AR14 | | | | | | | | |
| Drugs for COVID-19 treatment | Anakinra | L04AC03 | | | | | | | | |
| Drugs for COVID-19 treatment | Lopinavir/ ritonavir | J05AR10 | | | | | | | | |
| Drugs for COVID-19 treatment | Baricitinib | L04AA37 | | | | | | | | |
| Drugs for COVID-19 treatment | Sarilumab | L04AC14 | | | | | | | | |
| Drugs for COVID-19 treatment | Colchicine | M04AC01 | | | | | | | | |
| Drugs for COVID-19 treatment | Ruxolitinib | L01XE18 | | | | | | | | |
| Drugs for COVID-19 treatment | Canakinumab | L04AC08 | | | | | | | | |
| Drugs for COVID-19 treatment | Tofacitinib | L04AA29 | | | | | | | | |
| Drugs for COVID-19 treatment | Heparin | B01AB | | | | | | | | |
| Hospital-used injectables | Anesthetics, general | N01A | | | | | | | | |
| Hospital-used injectables | Adrenergic and dopaminergic agents | C01CA | | | | | | | | |
| Hospital-used injectables | Muscle relaxants | M03AB, M03AC | | | | | | | | |
| Hospital-used injectables | Ascorbic acid | A11GA01 | | | | | | | | |
| Hospital-used injectables | Hypnotics and sedatives | N05C | | | | | | | | |
| Hospital-used injectables | Antidotes | V03AB | | | | | | | | |
| Hospital-used injectables | Antithrombotic agents | B01AD | | | | | | | | |
| Drugs for non-prescription-based out-of-pocket supply | Anxiolytics | N05BA | | | | | | | | |
| Drugs for non-prescription-based out-of-pocket supply | Vitamin D and analogues | A11CC | | | | | | | | |
| Drugs for non-prescription-based out-of-pocket supply | Drugs used in erectile dysfunction | G04BE | | | | | | | | |
| Drugs for non-prescription-based out-of-pocket supply | Non-Steroidal Anti-Inflammatory Drug, Paracetamol | N02B | | | | | | | | |

© 2021 Ammassari A et al. *JAMA Network Open.*
